# The effect of Humanitude care methodology on improving empathy: a six-year longitudinal study of medical students in Japan

**DOI:** 10.1186/s12909-021-02773-x

**Published:** 2021-06-04

**Authors:** Yusuke Fukuyasu, Hitomi U. Kataoka, Miwako Honda, Toshihide Iwase, Hiroko Ogawa, Masaru Sato, Mayu Watanabe, Chikako Fujii, Jun Wada, Jennifer DeSantis, Mohammadreza Hojat, Joseph S. Gonnella

**Affiliations:** 1 Department of Nephrology, Rheumatology, Endocrinology and Metabolism, Okayama University Graduate School of Medicine, Dentistry and Pharmaceutical Sciences, Okayama, Japan; 2 Okayama University Hospital Center for Diversity and Inclusion, 2-5-1 Shikata-cho, Kita-ku, Okayama, 700-8558 Japan; 3 Department of Primary Care and Medical Education, Okayama University Graduate School of Medicine, Dentistry and Pharmaceutical Sciences, Okayama, Japan; 4 Geriatric Research Division, National Hospital Organization Tokyo Medical Center, Tokyo, Japan; 5 Department of Psychiatry and Human Behavior, Asano-Gonnella Center for Research in Medical Education and Health Care, Sidney Kimmel Medical College at Thomas Jefferson University, Philadelphia, PA USA

**Keywords:** Empathy, Humanitude, Medical education

## Abstract

**Background:**

Empathy, which involves understanding another person’s experiences and concerns, is an important component for developing physicians’ overall competence. This longitudinal study was designed to test the hypothesis that medical students’ empathy can be enhanced and sustained by Humanitude Care Methodology, which focuses on perception, emotion and speech.

**Methods:**

This six-year longitudinal observational study examined 115 students who entered Okayama University Medical School in 2013. The study participants were exposed to two empathy-enhancing programs: (1) a communication skills training program (involving medical interviews) and (2) a Humanitude training program aimed at enhancing their empathy. They completed the Jefferson Scale of Empathy (JSE) seven times: when they entered medical school, before participation in the first program (medical interview), immediately after the first program, before the second program (Humanitude exercise), immediately after the second program, and in the 5th and 6th year (last year) of medical school. A total of 79 students (69% of the cohort) completed all seven test administrations of the JSE.

**Results:**

The mean JSE scores improved significantly after participation in the medical interview program (*p* < 0.01) and the Humanitude training program (*p* = 0.001). However, neither program showed a sustained effect.

**Conclusions:**

The Humanitude training program as well as medical interview training program, had significant short-term positive effects for improving empathy among medical students. Additional reinforcements may be necessary for a long-term sustained effect.

## Background

Empathy, the ability to view experiences from another person’s point of view [1], is considered an important component of the clinicians’ overall competence [2] and an important quality for being “a good doctor” [3]. Empathy, in the context of patient care, has been defined as a predominantly cognitive (rather than emotional) attribute which involves an understanding (rather than feeling) of the patient’s sufferings, concerns and perspectives, with a capacity to communicate this understanding, and an intention to help the patient [2].

Past studies have shown that physicians’ empathy is positively and significantly associated with clinical outcomes among patients with diabetes [4, 5]. Various approaches and targeted educational programs have been implemented in order to enhance the empathy of medical students and physicians [2, 6] including the following: exposure to role models, audio- or video-taping of patient encounters, improving interpersonal skills, role playing (e.g. aging game), being a patient navigator (shadowing patients to offer them help), hospitalisation experiences, theatrical performances, studying literature and the arts, improving narrative skills and reflective writing, and utilising the Balint method (meeting in a small group to discuss cases that may be considered difficult, particularly regarding physician-patient relationships).

### Humanitude training and empathy

Our previous study [7] showed that a targeted educational program in communication skills training (medical interview) significantly improved empathy; however, we found that this empathy enhancement effect was not sustained. Therefore, in the current study, along with communication skills training, we introduced multimodal comprehensive communication (Humanitude) training to test whether this would result in an enhanced and sustained empathy.

In 1979, Gineste and Marescotti developed the Humanitude Care Methodology, a care methodology involving multimodal comprehensive communication, which focused on perception, emotion, and speech [8]. Humanitude has been implemented in a wide range of patient care contexts from paediatrics to geriatrics. In dementia care, it has been considered especially effective for dealing with the many challenges of providing care for patients with behavioural and psychological symptoms of dementia (BPSD). When used in this setting, the Humanitude approach focuses on non-verbal communication forms because people with dementia may not necessarily have the ability to communicate verbally.

Humanitude is based on four basic pillars: Gaze, Speech, Touch, and Verticality.

What follows are some basic explanations of Gaze, Speech, Touch and Verticality.

#### Gaze

With Gaze, caregivers keep their eye contact with patients closely and horizontally. Eye contact generally implies liking and preference, although there are important exceptions. When displeasure cues (e.g. words, bodily tension) accompany eye contact, they imply a strong negative attitude. Eye contact essentially implies communicator arousal and, depending on its association with pleasant and unpleasant verbal or non-verbal cues, serves to intensify the communication of positive versus negative attitudes [9]. In Humanitude, distance, angle of sight and duration of eye contact are key features of gazing for establishing positive relationships between caregivers and care receivers.

#### Speech

With Speech, caregivers continually speak to the patients while caring for them with positive words in slow and lower-tuned pitch, even if the patients do not respond or have no ability to speak. In Humanitude, speech is not limited to verbal communication. Along with the spoken words, the voice also conveys non-verbal information, including pitch, speed, volume, and vocabulary, which express emotional information.

#### Touch

The ideal touch, as utilised by caregivers, is characterised as being wide, slow, and gentle; for instance, caregivers should hold onto a patient’s arm by supporting it softly rather than by grasping the patient’s wrist. The oldest skill in medicine is the physician’s laying hands on the patient [10]. Therefore, touching during physical examinations has traditionally been regarded as the opening gate to the diagnosis and sometimes to therapeutic benefits. Touching is not only reminiscent of maternal stroking that generates a feeling of security, but also conveys affection and empathic support [2, 11]. As a non-verbal communication, the touching skills of Humanitude put emphasis on the strength, width, pressure and speed of touch, and also put Penfield’s somatosensory homunculus [12] into consideration. When caregivers apply touch to the patient, they start with less sensitive areas (for example, back and arms).

#### Verticality

Verticality refers to helping patients achieve a standing-up position. Standing up builds up several physiological merits related to organs and tissues (e.g., gravity helps to prevent osteoporosis and maintains muscle strength, while the act of standing up improves blood circulation), but its effects are not just physiological [13]. The act of standing up enables patients to recognise the space between themselves and others more easily compared to when they are bedridden, and this allows patients to develop feelings of human dignity by recognising human relationships in society.

Together, these four pillars can convey various positive emotions to care receivers. They are operationalised and systematised in order to be replicated in different contexts, and there are now about 150 techniques based on a relational premise, which promote the professionalisation of caregiver-patient relationships [14].

### Study purpose

In 2015, Okayama University Medical School introduced Humanitude into its regular curriculum in an effort to develop student empathy and patient care skills. In Japan, Okayama University Medical School became the second medical school to introduce Humanitude into its regular medical education curriculum. We designed this six-year longitudinal study to examine whether providing Humanitude training to these Japanese medical students resulted in enhanced and sustained empathy. We tested the following hypotheses:

1. Medical students’ empathy can be enhanced through Humanitude training.
2. Humanitude training (which focuses more on non-verbal communication training) will enhance responses to JSE items pertaining to body language and non-verbal communication more than medical interviews with standardised patients (which focus more on verbal communication skills training).
3. The empathy enhancement resulted from the Humanitude training can have sustained effect.

## Methods

The participating cohort in this six-year longitudinal study, approved by the IRB of Okayama University, included 115 medical students (76% men, *n* = 88) who entered Okayama University in 2013. Okayama University is a national university in Okayama, Japan, and has 12 schools in addition to the medical school.

### Instrument

We used the Japanese translation of the Student-Version (S-Version) of JSE in this study. The JSE scale is a 20-item validated instrument for measuring empathy in the context of patient care in medical students, other health professions students, and practitioners in the health professions including physicians. Each item is answered on a 7-point Likert scale (1 = strongly disagree, 7 = strongly agree). Evidence in support of the JSE’s construct validity [2, 15]; criterion-related validity [16, 17]; predictive validity [18]; internal consistency reliability [15, 17]; and test-retest reliability [15] has been reported. The Japanese translation of the JSE was performed by Kataoka et al., and the construct validity and reliability of its psychometrics in Japanese medical students have been confirmed [19].

### Procedures

Medical student participants underwent a medical interview workshop (Intervention 1) during Year 2 or 3, and the Humanitude training (Intervention 2) during Year 4. The JSE was administered during Year 1 (baseline), before Intervention 1 (pre-test) during Year 2 or 3, and after Intervention 1 (post-test) during Year 2 or 3. During Year 4, the JSE was administered before Intervention 2 (pre-test), and after Intervention 2 (post-test). During Year 5, the JSE was administered after the Objective Structured Clinical Examination (OSCE). Finally, during Year 6, the JSE was administered after students took the medical school graduation examination.

#### Intervention 1 (medical interview)

A special four-hour training medical interview workshop was offered with standardised patients (SPs) and designed to enhance empathy among medical students. The workshop was mandatory, and participating cohort of students attended one of the three workshops offered (maximum capacity per workshop = 40).

Each workshop was conducted over a single day and included the following sections: (1) a lecture about communication and medical interviewing; (2) orientation about the next session; (3) role playing as a student doctor with SPs in medical interviewing sessions; and (4) feedback, discussion, and summary of the workshop.

In the third section of the workshop, the participating students were divided into smaller subgroups of about 8 students each. Each subgroup then used its own simulated examination room for five different medical interview sessions, and five different standardised patients with different chief complaints took turns entering the simulated exam room. For each SP, one or two medical students played the role of a doctor, and conducted a medical interview with the SP for 10 min, while other students in their subgroup observed and assessed the interview. A detailed description of the workshop on medical interview is reported elsewhere [7].

#### Intervention 2 (multimodal comprehensive communication training: Humanitude training)

Training on Humanitude was given to medical students over 2 days in Year 4 as a mandatory program. The 8-h Humanitude training program for medical students consisted of an orientation lecture (60 min), workshops (360 min), and a review of the workshops (60 min), which was designed to enhance empathy among medical students. Workshops included three components: (1) Basic concept of Humanitude (60 min): lecture and discussion; (2) Four pillars (60 min each) Gaze, Touch, Speech, and Verticality: lecture and discussion and an exercise regarding each pillar; and (3): Summary and implication of Humanitude with regard to clinical situations (60 min): lecture and discussion. The class had three official instructors and four supporters to assist the instructors.

The program started with an interactive lecture on Humanitude’s philosophy, communication skills, and the stepwise sequence of care. After the lecture, small group workshops were offered for the four basic pillars of Humanitude, so that the students could experience the multimodal comprehensive communication of Humanitude.

The Humanitude workshops included several opportunities for practicing the four basic pillars: Gaze, Speech, Touch, and Verticality. This training aimed to help students learn the four basic pillars and emphasised the importance of not only verbal but also non-verbal communication. For example, in the Gaze workshop, students were asked to pair with a classmate and gaze at each other’s eyes for 1 min from a normal distance; then, they were asked to gaze at each other from a very close distance for 1 min. In the workshop on Speech, students were asked to talk to each other to try to convey positive emotions using the skills presented. In the workshop on Touch, students tried to touch each other’s backs as instructed in the lecture. In the workshop on Verticality, they learned how to assist standing up. Finally, students practiced using these pillars simultaneously, which is called multimodal communication.

### Testing

In all test administrations of the study, students were reminded that their responses on the assessment test (JSE) would be totally voluntary, would be kept strictly confidential, would not affect their academic record, and might be used as aggregated data for statistical analyses.

In order to assess the effect of the non-verbal communication aspect of Humanitude on the empathy scale, we conducted item analysis of the JSE. Two items of JSE mention body language and non-verbal cues: #4. “Understanding body language is as important as verbal communication in health care provider-patient relationships”; and #13. “Health care providers should try to understand what is going on in their patients’ minds by paying attention to their non-verbal cues and body language.” We analysed these two items individually to determine whether empathy improvement in the domain of non-verbal communication was statistically significant following the Humanitude lecture and workshops.

### Data analysis

Analysis of Variance (ANOVA) for a repeated measures design was used for statistical analyses, followed by t-test for repeated measures (t-test for paired samples) to test the statistical significance of differences in changes in empathy scores between the pre- and post-intervention. We also calculated Cohen’s *d* to estimate the effect size of the differences in mean scores before and after the training program in order to determine the practical (clinical) significance of the analysis findings. The effect size estimates were determined as follows: about 0.20 was considered negligible, around 0.50 was moderate, and around 0.80 was large [20, 21]. Statistical analyses were carried out using the Statistical Package for the Social Sciences (SPSS), version 26.0 (IBM, Armonk, New York).

## Results

Of the total students in the class (*n* = 115), 113 (98%) completed the JSE during Year 1; 107 (93%) completed the pre-test during Year 2 or 3 and 110 (96%) completed the post-test during Year 2 or 3; 109 completed (95%) the pre-test during Year 4 and 108 (94%) completed the post-test during Year 4; during Year 5, 97 (84%) completed the JSE, and during Year 6, 89 (77%) completed the JSE.

**Fig. 1:**
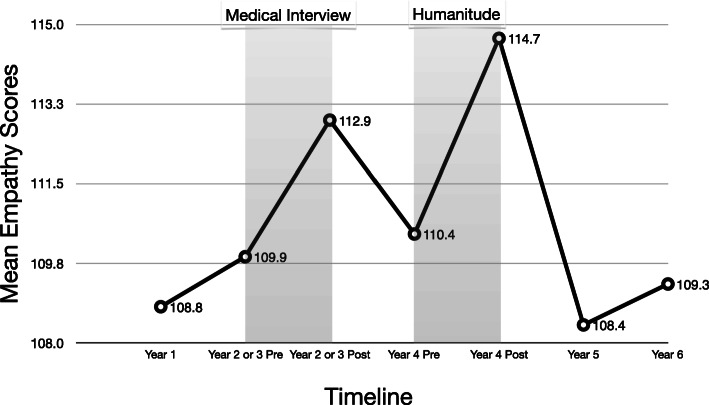
Changes in mean JSE scores before and after participation in medical interview and Humanitude programs

The final analysis included 79 students with complete data, who represented 69% of the total cohort (72% men, *n* = 57). Means and standard deviations of the total JSE score, and summary results of statistical analyses appear in Table 1 and Fig. 1. The total pretest mean JSE score was 109.9 (*SD* = 11.9) prior to participation in the program, which significantly increased to 112.9 (*SD* = 12.3) immediately after the completion of Intervention 1 (Medical Interview) (*t* = 2.65, *p* < 0.01, *r* = 0.668, effect size estimate *d* = 0.25), and significantly increased to 114.7 (*SD* = 14.3) immediately after the completion of Intervention 2 (Humanitude) (*t =* 3.31, *p* = 0.001, *r* = 0.666, effect size estimate *d* = 0.30). However, the mean JSE score reverted to the baseline level during Years 5 (*M* = 108.4, *SD* = 15.0) and 6 (*M* = 109.3, *SD* = 14.3) of medical school, indicating that the empathy enhanced during the programs (in Years 3 and 4) was not sustain over a long term (during Years 5 and 6).

**Table 1:** Means and Standard Deviations of the JSE scores before and after medical interview and Humanitude

	Total JSE Score		
Test Administration	*M*	*SD*	*t-*test
Year 1 (baseline)	108.8	12.9	
Pre-test before intervention 1 (Medical Interview)	109.9	11.9	2.65**
Post-test after intervention 1	112.9	12.3	
Pre-test before intervention 2 (Humanitude)	110.4	14.3	3.31***
Post-test after intervention 2	114.7	14.3	
Year 5	108.4	15.0	
Year 6	109.3	14.3	

Total JSE Score: *F* (6, 468) =5.32 *p* < 0.0001 *d* = 0.52

**p < 0.01, ***p = 0.001

**Fig. 2:**
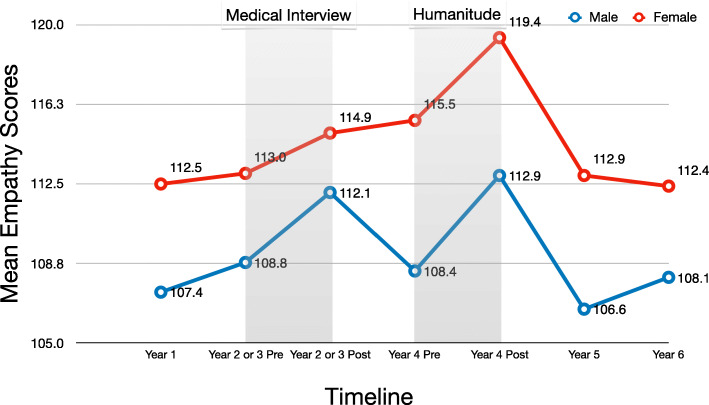
Changes in mean JSE scores before and after medical interview and Humanitude training by gender

Due to gender differences in empathy, we conducted additional analyses of mean differences based on gender (Fig. 2). Male students’ mean empathy scores significantly improved with both Medical Interview and Humanitude interventions (Medical Interview: *t* = 2.54, *p* < 0.05, effect size estimate *d* = 0.34; Humanitude: *t* = 2.60, *p* < 0.05, effect size estimate *d* = 0.34), whereas female students’ mean empathy scores improved significantly with Humanitude (*t* = 2.84, *p* < 0.001, effect size estimate *d* = 0.60) but not with Medical Interview (*t* = 0.91, *p* = 0.37, effect size estimate *d* = 0.19).

**Fig. 3:**
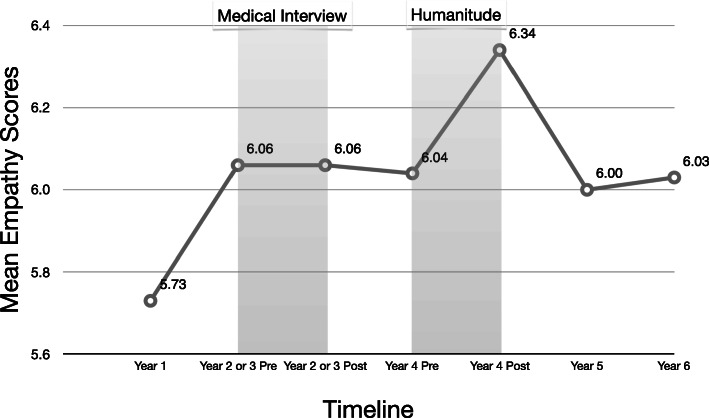
Changes in JSE scores (Item #4) before and after participation in medical interview and Humanitude. Humanitude *t* = 3.49, *p* < 0.001, Effect size estimate *d* = 0.28

Means and standard deviations of Item 4 and Item 13 of the JSE in different periods prior to and after Intervention 1 (medical interview) and Intervention 2 (Humanitude) are reported in Table 2. JSE Item 4 (Understanding body language is as important as verbal communication in health care provider-patient relationships.) significantly improved with Humanitude program but did not change with Medical Interview program (Fig. 3). However, JSE Item 13 (Health care providers should try to understand what is going on in their patients’ minds by paying attention to their non-verbal cues and body language.) was not associated with statistically significant improvements in either the Medical Interview or the Humanitude program.

**Table 2:** Means and Standard Deviations of Item 4 and Item 13 of the JSE

	Item 4			Item 13		
Test Administration	*M*	*SD*	*t-*test	*M*	*SD*	*t-*test
Year 1 (baseline)	5.73	1.48		6.18	1.15	
Pre-test before intervention 1 (Medical Interview)	6.06	1.05	0.00^NS^	6.22	0.81	0.99 ^NS^
Post-test after intervention 1	6.06	0.90		6.32	0.86	
Pre-test before intervention 2 (Humanitude)	6.04	1.08	3.49***	6.16	1.07	1.17 ^NS^
Post-test after intervention 2	6.34	0.88		6.29	0.85	
Year 5	6.00	1.16		6.03	1.00	
Year 6	6.03	1.11		6.08	0.97	

***p < 0.001, NS: not significant

## Discussion

The present study focused on testing its three hypotheses with regard to the effects of Humanitude training on enhancing and sustaining empathy among medical students. Our findings confirmed our first research hypothesis regarding the effect of Humanitude training on enhancing empathic orientation in patient care. Furthermore, we found that the produced empathy enhancement was more statistically significant immediately after the Humanitude program rather than after the Medical Interview program. The effect size estimates regarding empathy improvement were in the small to medium range (0.25 for the Medical Interview and 0.30 for the Humanitude program). This suggests that both programs produced empathy enhancement that was not negligible. The second hypothesis confirmed that Humanitude training produced more pronounced changes in items pertaining to body language on the empathy measuring instrument because, compared to the medical interviewing, it focused more on non-verbal clues. While Humanitude includes a “speech” element, the communication of Humanitude does not necessarily have to be mutual; for instance, elderly bedridden patients with cognitive impairments may not necessarily respond to their caregivers. However, medical interviews are predicated on mutual conversation, and without a verbal element, it is difficult to continue the interviewing process. Focusing on non-verbal cues requires more attention and effort from both caregivers and patients, which may contribute towards enhancing their cognitive understanding of each other, thus improving empathy in the relationship.

Item analyses for the JSE Items 4 and 13 suggested that the empathy factor enhanced by Humanitude training could differ from that enhanced by Medical Interview. The JSE Item 4 (Understanding body language is as important as verbal communication in health care provider-patient relationships.), which emphasises the importance of body language and non-verbal communication, showed significant enhancement only after the Humanitude training and not after the Medical Interview program. Meanwhile, the JSE Item 13 (Health care providers should try to understand what is going on in their patients’ minds by paying attention to their non-verbal cues and body language.), did not show any significantly enhancement after either program. The results regarding JSE Item 4 could be attributed to the possibility of students realising the importance of non-verbal communication by directly understanding the effectiveness of gaze and touch from their own experiences in the Humanitude intervention.

Our third hypothesis, concerning the sustained effect of Humanitude training, was not confirmed. This finding was consistent with the results of a previous study [7]. These results indicated that, if it is to be sustained, empathy enhancement may require additional reinforcement [22]. Chen et al. reported a sustained effect on empathy for at least one and a half years after a two-month long narrative medicine program was implemented [23]. Additional remedial programs are necessary for reinforcing the importance of empathy in patient care during medical education, to sustain improvement in empathy among medical students, and to avoid reverting the improved empathy score to the pre-intervention score.

In the United States, various longitudinal [24], and cross-sectional [25] studies have shown a significant decline in empathy among third-year medical students (equivalent to fifth-year medical students in Japan). In contrast, one cross-sectional study conducted in Japan showed no such decline during medical school [19]. This is consistent with previous [7] and present six-year longitudinal studies, where the empathy scores reverted to the baseline level during Year 5, but a significant decline below the baseline level was not observed.

We observed that the empathy score during Year 2 or 3 before Intervention 1 was higher than that during Year 1 and that the empathy score decreased during Year 4 (before Intervention 2). We collected the data for Year 1 when the students started the medical school, and during Year 2 or 3 (at the end of the second year or during the first half of the third year); the data were collected after the implementation of various lectures on liberal arts and practical programs in behavioural sciences during Years 1 and 2. This may have resulted in a slight increase in the Year 2 or 3 pre-intervention scores. However, the increased scores after Intervention 1 dropped to baseline scores during Year 4. One reason could be that the medical interview session only had a temporal effect. This reversion could also be attributable to their curriculum. Students have a very tight schedule that only focuses on the scientific side of medicine during Year 3, and the first half of Year 4 consists mainly of classroom lectures. Thus, student do not have the opportunity to reinforce their empathy within this curriculum. From Years 1 to 4, we collected the relevant data before the ordinal classes, and the students were not in a particularly stressed state. However, during Years 5 and 6, these relevant data were collected just after they finished their examinations, as they did not have any ordinal classes. During this period, the participating students may have been in an especially distressed state and may have even lacked sufficient sleep. We speculate that their empathy may have been affected by their difficult curricula and challenging environments.

Even during Year 4, female students’ scores did not change, and male students’ scores decreased; the mean score slightly decreased, as shown in Fig. 2. A previous study [23] also suggested that female students’ scores are more likely to be maintained, and male students’ scores are not.

Furthermore, we observed a high response rate (93 to 98%) for the JSE during Years 1 to 4. However, this response rate dropped to 84% during Year 5 and 77% during Year 6. Thus, Japanese students may have a tendency to complete most of the questionnaire items, even if they are optional. This high response rate could be attributed to the fact that the students answered the JSE during class from the first to fourth years, whereas during the fifth and sixth years, as we mentioned above, they answered it just after their examinations. This may explain the drastic drop in the response rate during Years 5 and 6.

Noteworthily, our findings had some limitations because this current research was conducted at a single institution. Thus, the study’s findings had limited generalisability. The study may thus have to be replicated in other medical schools in order to gain a larger sample size and consequently increase its statistical power. Furthermore, the study did not utilise a control group, which could be compared to those who participated in the Medical Interview and Humanitude training programs. Future research could increase the accuracy of this study’s data by utilising a control group to confirm the causal link between program participation and enhanced empathy.

## Conclusions

Medical students’ empathy can be enhanced through Humanitude training. Since Humanitude emphasises non-verbal communication, it may be better at enhancing different factors related to empathy than medical interviews with SPs, which emphasise verbal communication. However, the empathy enhancement was not sustained over a long period, and additional reinforcements, including Humanitude training, medical interviews, and other educational programs, may be necessary for meaningfully enhancing and possibly sustaining medical students’ empathy.

## Data Availability

The datasets generated and/or analysed during the current study are not publicly available because informed consent did not include the permission to disclose the raw data of students’ performances.

## Abbreviations

JSE: Jefferson Scale of Empathy
BPSD: Behavioural and psychological symptoms of dementia
OSCE: Objective structured clinical examination
SPs: Standardised patients
